# Analysis of Patients with Severe ARDS on VV ECMO Treated with Inhaled NO: A Retrospective Observational Study

**DOI:** 10.3390/jcm13061555

**Published:** 2024-03-08

**Authors:** Stefan Muenster, Jennifer Nadal, Jens-Christian Schewe, Heidi Ehrentraut, Stefan Kreyer, Christian Putensen, Stefan Felix Ehrentraut

**Affiliations:** 1 Department of Anesthesiology and Intensive Care Medicine, University Hospital Bonn, 53127 Bonn, Germany; 2 Institute of Medical Biometry, Informatics and Epidemiology, University Hospital Bonn, 53127 Bonn, Germany; 3 Department of Anesthesiology, Intensive Care Medicine and Pain Therapy, University Medical Center, 18057 Rostock, Germany

**Keywords:** inhaled nitric oxide, severe ARDS, VV ECMO, pulmonary hypertension, inhaled nitric oxide responder

## Abstract

(1) **Background**: This retrospective study focused on severe acute respiratory distress syndrome (ARDS) patients treated with veno-venous (VV) extracorporeal membrane oxygenation (ECMO) and who inhaled nitric oxide (NO) for pulmonary arterial hypertension (PAH) and/or right ventricular failure (RV failure). (2) **Methods**: Out of 662 ECMO-supported patients, 366 received VV ECMO, including 48 who inhaled NO. We examined the NO’s indications, dosing, duration, and the ability to lower PAH. We compared patients with and without inhaled NO in terms of mechanical ventilation duration, ECMO weaning, organ dysfunction, in-hospital mortality, and survival. (3) **Results**: Patients received 14.5 ± 5.5 ppm NO for 3 days with only one-third experiencing decreased pulmonary arterial pressure. They spent more time on VV ECMO, had a higher ECMO weaning failure frequency, and elevated severity scores (SAPS II and TIPS). A Kaplan–Meier analysis revealed reduced survival in the NO group. Multiple variable logistic regression indicated a twofold increased risk of death for ARDS patients on VV ECMO with NO. We observed no increase in continuous renal replacement therapy. (4) **Conclusions**: This study suggests that persistent PAH and/or RV failure is associated with poorer outcomes in severe ARDS patients on VV-ECMO, with an inhaled NO responder rate of only 30%, and it does not impact acute kidney failure rates.

## 1. Introduction

Inhaled nitric oxide (NO) has been widely used as a selective pulmonary vasodilator to treat pulmonary arterial hypertension (PAH) and/or right ventricular failure (RV failure) [1]. Inhaled NO is distributed only to ventilated lung regions and exerts the unique ability of selectively inducing smooth muscle relaxation in the pulmonary vasculature in said regions [1,2]. Thus, inhaled NO improves arterial blood oxygenation, decreases intrapulmonary shunting, and enhances blood flow distribution toward the ventilated alveolar regions of the lungs [2,3]. The high affinity of NO to oxy-hemoglobin in red blood cells leads to the rapid formation of nitrate and met-hemoglobin; this mechanism limits the vasodilation effects to the pulmonary vasculature and thereby avoids systemic arterial hypotension [4,5]. Inhaled NO concentrations of up to 80 parts per million (ppm) have been administered safely without unfavorable systemic side effects [6,7,8].

Acute respiratory distress syndrome (ARDS) is an acute diffuse, inflammatory lung injury, leading to substantial loss of aerated lung tissue and is clinically characterized by severe hypoxemia and bilateral radiographic opacities [9,10,11,12]. Most of the frequent etiologies of ARDS include pneumonia, sepsis, and trauma [9,10,11,12]. In addition, ARDS represents 10% of all intensive care unit admissions and 23% of all patients requiring mechanical ventilation [13]. The mortality rates in patients with ARDS range from approximately 25% to 46% across all severities and can be even higher when associated with the dysfunction of other organs [13,14,15,16,17,18,19].

Patients suffering from ARDS frequently present with PAH, whereas increased intrapulmonary shunting caused by the perfusion of non-aerated alveoli contributes to severe arterial hypoxemia [20,21]. Moreover, elevated pulmonary artery pressure raises transcapillary pressure and thereby increases the risk of alveolar edema, which may aggravate the ARDS [22,23]. In addition, PAH may lead or contribute to RV failure and is an independent risk factor for mortality in patients with ARDS [24,25]. Given its pharmacological properties, inhaled NO may lower pulmonary arterial pressure, thereby reducing the risk of RV failure and intrapulmonary shunting [26,27,28]. Although randomized controlled trials (RCTs) on inhaled NO use have shown both improved oxygenation and hemodynamics in the acute phase of ARDS in adults, all studies thus far have failed to demonstrate any clinically significant benefit of inhaled NO on survival or ventilator-free days [29,30].

Veno-venous extracorporeal membrane oxygenation (VV ECMO), widely considered a life-saving procedure, may restore oxygenation and eliminate carbon dioxide accumulation when conventional mechanical ventilation fails to ensure a sufficient gas exchange in severe ARDS [11,31,32].

Patients with severe ARDS and VV ECMO support with concomitant inhaled NO administration represent a very specific and limited cohort. Data that elucidate the use of inhaled NO in this specific cohort are lacking, and little is known about survival and mortality in this population. To gain more insight into this specific and limited group of critically ill patients, we performed a retrospective observational study of patients with severe ARDS and VV ECMO support treated with inhaled NO as rescue therapy.

## 2. Materials and Methods

### 2.1. Study Design

This retrospective observational study was conducted following the Strengthening the Reporting of Observational Studies in Epidemiology statement (STROBE).

### 2.2. Objectives

The objectives were as follows:

- To describe inhaled NO treatment in terms of the indication, dosing, and duration of application.
- To measure the inhaled NO ability to lower mean pulmonary arterial pressure (mean PAP) in patients under VV ECMO support who are responders and non-responders to inhaled NO.
- To describe the clinical characteristics, such as time on mechanical ventilation, weaning from VV ECMO, and organ dysfunction, as described by the sequential organ failure assessment (SOFA) score [33] and the therapeutic intervention scoring system (TISS) [34].
- To measure outcome parameters, such as survival in ICU, using the simplified acute physiology score II (SAPS II) [35], in-hospital mortality, and long-term survival.

### 2.3. Study Population

Data on all ECMO patients treated in the intensive care unit of the Department of Anesthesiology and Intensive Care Medicine at the quaternary level of the University Hospital, Bonn, Germany, between May 2015 and May 2021 were collected.

### 2.4. Inclusion Criteria

The inclusion criteria included the following:

- Age > 18 years old;
- Electronic medical records available, including VV ECMO run parameters, vital parameters, and laboratory measurements (both point-of-care and laboratory diagnostics);
- VV ECMO support;
- ARDS following the Berlin definition [11], and with or without inhaled NO administration.

### 2.5. Exclusion Criteria

The exclusion criterion was veno-arterial ECMO support.

### 2.6. Indication for ECMO

Indications for VV ECMO support complied with the Extracorporeal Life Support Organization General Guidelines [36]. Indications included the treatment of severe hypoxemia and hypercapnia and the prevention of possible harmful mechanical ventilation (i.e., prolonged use of exceedingly high peak inspiratory pressures or driving pressure > 15 cmH_2_O) to ensure sufficient gas exchange according to ARDS network definitions [16]. All decisions for initiating VV ECMO support were based on the consensus between at least two experienced senior critical care physicians of the ARDS/ECMO team of the current study.

### 2.7. Indication for Inhaled NO Delivery and Definition of PAH and RV Failure

Inhaled NO (NO-A nitric oxide delivery system, EKU Elektronik GmbH, Leiningen, Germany) was administered at the treating physician’s discretion after bedside evaluations of pulmonary hemodynamics and right heart function, as indicated by invasive pulmonary hemodynamics (assessed using a Swan–Ganz catheter) and/or transesophageal echocardiography. The use of pulmonary arterial catheters and transesophageal echocardiography are standard care procedures at our institution in this specific patient population. A mean PAP ≥ 25 mmHg was used as a cut-off value for inhaled NO application [37]. A positive response to inhaled NO was determined as a mean PAP reduction of ≥6 mmHg following the administration of inhaled NO within 30 min. Other indicators, such as pulmonary vascular resistance or the cardiac index, were not used due to insufficient data regarding their validity during extracorporeal life support.

In the present study, RV failure was defined by transesophageal echocardiography, as suggested by Vieillard-Baron et al. [38]. In severe ARDS, acute cor pulmonale or severe RV dilatation accurately reflects RV failure, particularly when right atrial pressure is increased.

### 2.8. Ethics

Ethical approval for this study (Ethical Committee N° 492/20) was provided by the Ethical Committee of the University Hospital Bonn, Bonn, Germany (Chairperson Prof. K. Racké) on the 6th November 2020, and the need for informed consent was waived.

### 2.9. Statistical Analyses

All data are presented as the median and interquartile range (IQR) for non-normally distributed variables or mean ± standard deviation for normally distributed, continuous variables, as appropriate, and frequency distributions with percentages for categorical variables. The Wilcoxon rank-sum test was used to analyze group differences in non-normally distributed variables. Nominal variables were assessed using Fisher’s exact test or Pearson’s Chi-square test. Moreover, a multiple variable logistic regression analysis was performed, with in-hospital demise as the dependent variable and the following independent variables: inhaled NO during VV ECMO, age, BMI, and SOFA score at day 0 of VV ECMO implantation. Variables were chosen based on clinical plausibility and through backwards elimination during model testing. Variables with a *p*-value of 0.05 were considered significant in the multivariable regression analysis.

The Kaplan–Meier method and the stratified log-rank test were performed to analyze survival [39]. All analyses were performed on R version 4.1.2 [40] All tests were two-sided, and *p* < 0.05 was determined as the cut-off for significance. No adjustments were made for multiple tests, and *p* values should be interpreted as exploratory only.

## 3. Results

### 3.1. Identification and Characteristics of the Eligible Study Cohort

**Figure 1:**
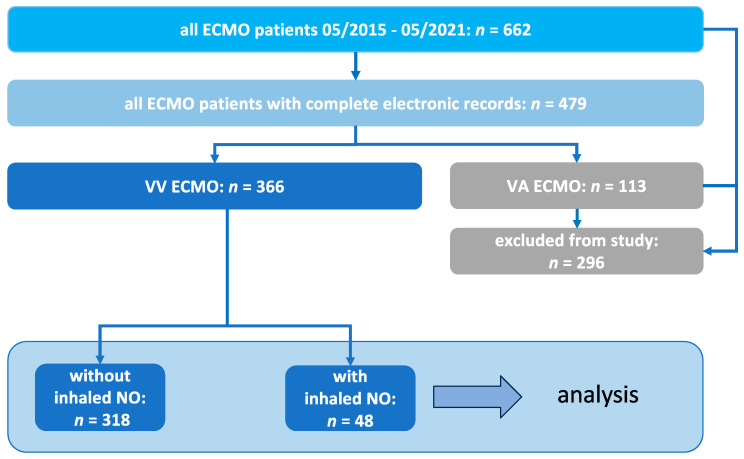
Inclusion process for the selected patients of the retrospective observational study.

To identify the eligible study cohort, 662 patients with ECMO were screened during the study period. Patients with complete electronic medical records were analyzed (Figure 1). The 366 patients who underwent VV ECMO support were identified as the eligible study cohort, which included 48 individuals with known PAH and/or RV failure, treated with inhaled NO as rescue therapy. This study cohort would be further analyzed and represents the group of interest for the retrospective observational analysis. Baseline and clinical characteristics are provided in Table 1 and Table 2.

### 3.2. Indication, Dosing, and Duration of Inhaled NO Treatment in Patients with VV ECMO

To evaluate whether patients with VV ECMO support may benefit from inhaled NO administration, transesophageal echocardiography, and/or invasive monitoring using a pulmonary artery catheter, were performed before the start of inhaled NO. Subsequently, three groups of indications were identified for the use of inhaled NO in patients undergoing VV ECMO (Table 1): PAH, RV failure, or a combination of both pathologies.

In addition, the inhaled NO doses administered in the VV ECMO patient cohort were investigated. Gaseous NO was administered at an average dose of 14.5 ± 5.5 ppm (ranging from a minimum dose of 6.9 ppm to a maximum of 20 ppm). The duration of treatment was 3 days (IQR, 1.76–4.41).

### 3.3. Ability of Inhaled NO to Lower Mean PAP in Responder and Non-Responder Patients

**Figure 2:**
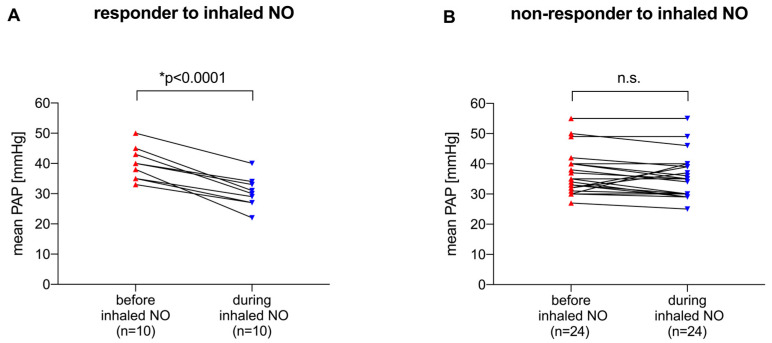
(**A**) Mean PAP before and during inhaled NO treatment in patients with ARDS under VV ECMO defined as inhaled NO responders (decrease in mean PAP during treatment ≥ 6 mmHg). (**B**) Mean PAP before and during inhaled NO treatment in patients with ARDS under VV ECMO defined as inhaled NO non-responders (decrease in mean PAP during treatment < 6 mmHg). Differences in mean PAP were compared using the paired *t*-test with statistical significance at *p* < 0.01. ppm: parts per million, inhaled NO: inhaled nitric oxide, mean PAP: mean pulmonary artery pressure.

As mentioned in the Methods section, a positive response to inhaled NO was defined as a mean PAP decrease ≥ 6 mmHg. In *n* = 34 patients, a Swan–Ganz catheter was inserted to continuously monitor pulmonary hemodynamics, allowing us to distinguish between inhaled NO responders and non-responders in these cases (in contrast to patients who were monitored discontinuously and solely via echocardiography). Responder patients with ARDS under VV ECMO showed a significant decrease in mean PAP when inhaled NO was administered (mean PAP before inhaled NO [39.4 ± 5.4 mmHg] vs. mean PAP during inhaled NO [30 ± 4.9 mmHg]; *p* < 0.0001; Figure 2A). In contrast, non-responder patients with ARDS did not show a significant decrease in mean PAP during inhaled NO treatment (mean PAP before inhaled NO [37.7 ± 7.8 mmHg] vs. mean PAP during inhaled NO [36.7 ± 8 mmHg]; Figure 2B).

**Figure 3:**
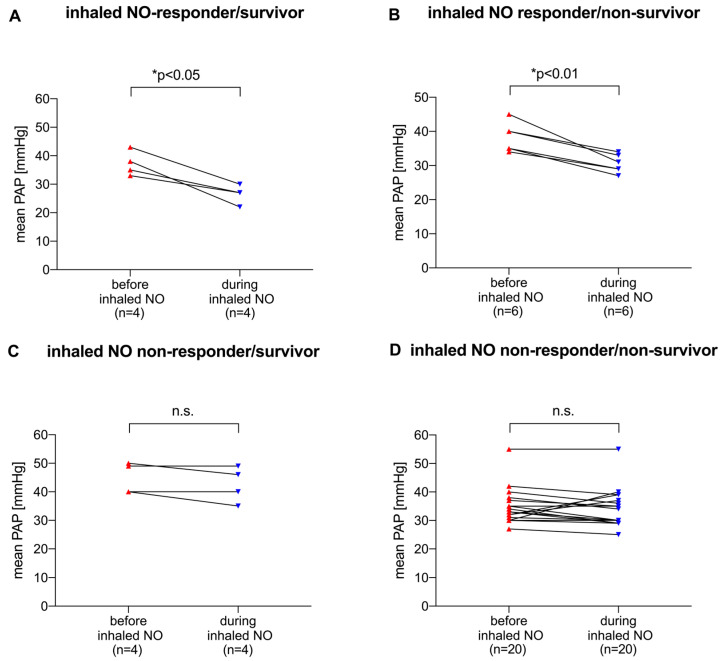
Ability of inhaled NO to reduce mean PAP in patients who respond to NO (**A**,**B**) and in patients who do not respond (**C**,**D**), and differentiation between survivors (**A**,**C**) and non-survivors (**B**,**D**). Differences in mean PAP were compared using the paired *t*-test with statistical significance at *p* < 0.05. ppm: parts per million, inhaled NO: inhaled nitric oxide, mean PAP: mean pulmonary artery pressure.

To analyze whether the ability of NO to lower pulmonary artery pressure in ARDS patients on VV ECMO would potentially affect survival, NO responder and non-responder patients were investigated in terms of survival depending on the pressure drop of the mean PAP (Figure 3). We found that NO-responder patients were scattered in the survivor (Figure 3A,C) and non-survivor (Figure 3B,D) subgroups. In addition, most patients belonged to the non-survivor cohort, regardless of NO responsiveness.

### 3.4. Organ Failure, ECMO Circuit Weaning, and Rate of Tracheostomy

Apart from the SOFA score on ICU admission, the severity of further organ failure was assessed using the SAPS II and TISS scores, both 24 h after ECMO initiation and at hospital discharge (Table 2). The SAPS II and TISS scores differed between the groups at hospital discharge, indicating an increased severity of illness in the inhaled NO group (SAPS II with inhaled NO: 57 (43–68) vs. SAPS II without inhaled NO: 46 (32–59), *p* = 0.0037, and TISS with inhaled NO: 30 (20–37) vs. TISS without inhaled NO: 22 (12–30), *p* = 0.0007, Table 2). The cardiopulmonary resuscitation rate before ECMO and the rate of sepsis, as defined by SEPSIS-3 [41] can be found in Table 2.

Weaning failure from VV ECMO support was observed in *n* = 35 (73%) inhaled NO-treated patients. The total time on mechanical ventilation was 22 days. The time on mechanical ventilation before VV ECMO, duration of VV ECMO, and rate of tracheostomy in inhaled NO-treated patients are reported in Table 2. Adjunctive therapies, i.e., continuous kidney replacement therapy and prone positioning before or during VV ECMO support, are also reported in Table 2.

### 3.5. Standard Ventilation Parameters and Blood Gas Analyses Both on Days 1, 3, and 7 during VV ECMO

To avoid the onset of PAH and/or RV failure, optimizing respiratory conditions in patients with ARDS is strongly recommended because a driving pressure ≥ 18 cmH_2_O, arterial partial pressure of carbon dioxide (P_a_CO_2_) ≥ 48 mmHg, and P_a_O_2_/F_I_O_2_ < 150 mmHg have been reported as risk factors [42]. A modified, simplified version of the ARDS Network’s lung-protective lower tidal volume strategy was applied to all patients in both cohorts as it was associated with low mortality rates in three previous ARDS Network trials (ARMA, ALVEOLI and FACTT) [16,43,44]. More specifically, standard PEEP strategies in patients with severe ARDS and VV ECMO support follow the higher PEEP/lower F_I_O_2_ table as shown in the ALVEOLI trial [43].

Ventilation parameters and arterial blood gas analyses were both recorded on days 1, 3, and 7 during VV ECMO with inhaled NO treatment (Supplemental Table S1). These findings showed no differences in minute ventilation, positive end-expiratory pressure (PEEP), or peak inspiratory pressure between the different days. Ventilation parameters, as well as arterial blood gas analyses, showed results that comply with the respective ARDS guidelines on mechanical ventilation [45].

### 3.6. Outcomes

**Figure 4:**
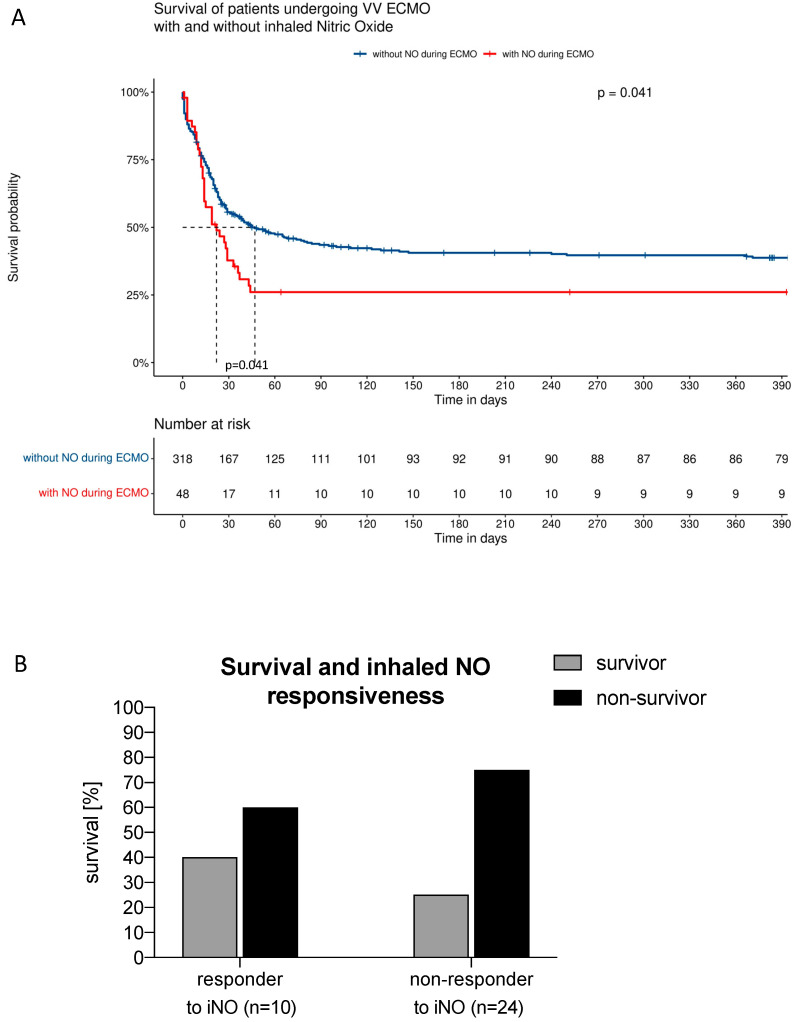
(**A**) Kaplan–Meier curve reporting the long-term survival rate (up to 390 days) of patients with severe ARDS on VV ECMO support with PAH and/or RV failure treated with inhaled NO. (**B**) Subgroup analysis of the survival rates of inhaled NO responders and non-responders.

**Figure 5:**
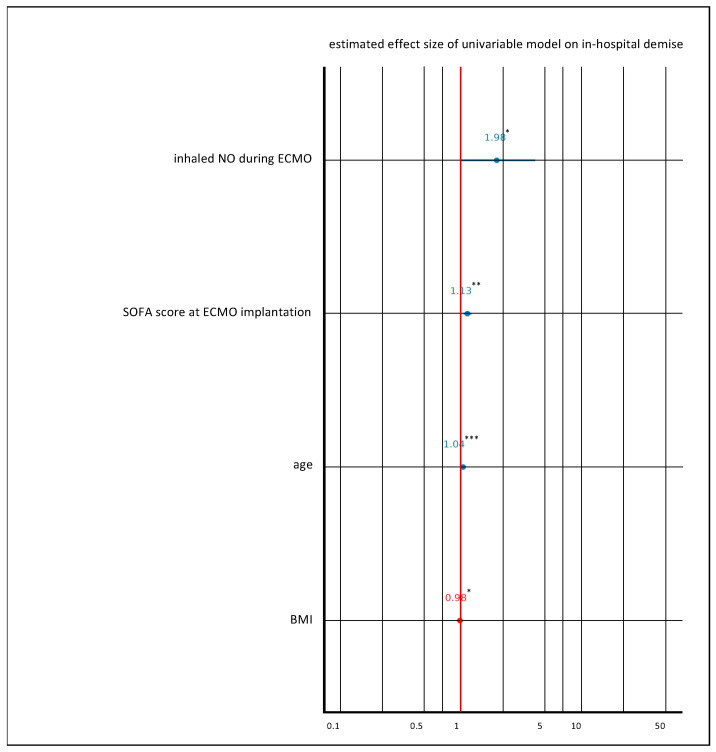
Multiple variable logistic regression analysis was performed, with in-hospital demise as the dependent variable and the following independent variables: inhaled NO during VV ECMO, age, BMI, and SOFA score at day 0 of VV ECMO implantation. Variables were chosen based on clinical plausibility and through backward elimination during model testing. Variables with a *p*-value of 0.05 (* *p* < 0.05, ** *p* < 0.01, *** *p* < 0.001) were considered significant in the multivariable regression analysis.

The Kaplan–Meier curve in Figure 4A shows the long-term survival rate (up to 390 days) of patients with severe ARDS on VV ECMO support with PAH and/or RV failure treated with inhaled NO. Long-term survival differed between the groups with a lower probability of survival in the inhaled NO group (Figure 4A, *p* = 0.041). Multiple variable logistic regression analysis revealed that the risk of death was twofold higher when inhaled NO was used during VV ECMO in patients with severe ARDS (odds ratio: 1.98 (1.00–4.12), * *p* < 0.05 Figure 5). In addition, the risk of death increased per year of patient age (odds ratio: 1.04 (1.02–1.06), *** *p* < 0.001, Figure 5), and per point of SOFA score (odds ratio: 1.13 (1.05–1.22), ** *p* < 0.01, Figure 5). In contrast, the BMI reduced the risk of death although the effect size is considered small (odds ratio: 0.98 (0.96–1.00), * *p* < 0.05, Figure 5).

To evaluate whether survival may depend on the ability of inhaled NO to lower an elevated mean PAP, a subgroup analysis of the fractional survival rates was performed in the inhaled NO responder and non-responder groups. Figure 4B shows that the survival in inhaled NO treated patients differed between inhaled NO responders (40%) and non-responders (25%), (*p* = 0.0341, Figure 4B).

Supplemental Table S2 presents the data on the length of ICU and hospital stay, in-hospital mortality rate, and median survival time of the study cohort. The known medical history indicated by the Charlson Comorbidity Index can be found in Supplemental Table S3.

## 4. Discussion

We investigated, in a retrospective observational study, patients with severe ARDS undergoing VV ECMO support with concomitant inhaled NO administration to gain more insight into this specific population. We analyzed clinical characteristics such as time on mechanical ventilation, weaning from VV ECMO, organ dysfunction, the inhaled NO treatment in terms of indication, dosing and duration, the ability of inhaled NO to lower PAP in responder and non-responder patients, the survival rate, in-hospital mortality, and long-term survival. We found that long-term survival was poor in patients with severe ARDS under VV ECMO support with persistent PAH and/or RV failure. A rescue therapy with the pulmonary vasodilator inhaled NO showed that only one-third of the cohort responded to the therapy with a sufficient decrease in the mean PAP. Intriguingly, survival between inhaled NO responders and non-responders showed that NO responder patients had a better survival rate. In general, patients that were treated with inhaled NO showed increased levels of illness severity at hospital discharge as indicated by the SPAS II and TISS scores. We did not find an increased rate of continuous kidney replacement therapy as marker for acute renal failure when inhaled NO concentrations were ≤20 ppm and when the treatment duration stayed ≤3 days.

Pulmonary vascular dysfunction is one of the pathophysiological hallmarks of ARDS that ultimately leads to a certain degree of PAH [46]. Recent data suggest that PAH and subsequent RV failure are medical burdens that occur in every second patient with moderate to severe ARDS and are independently associated with the risk of mortality [46,47]. In ARDS, multiple pathophysiological mechanisms that directly cause injury to pulmonary circulation include endothelial dysfunction, distal pulmonary vascular occlusion at the capillary level, pulmonary vasoconstriction, extrinsic vessel occlusion by alveoli distention, and, ultimately, vascular remodeling [48]. These mechanisms lead to increased pulmonary vascular resistance, precapillary pulmonary hypertension, and increased RV afterload [48]. The uncoupling between pulmonary circulation and the right heart ultimately leads to the breakdown of oxygen delivery.

Various strategies, including limiting volume loading and correcting blood pressure by infusing norepinephrine, have been suggested to decrease RV wall stress and RV end-diastolic pressure, thereby improving RV stroke volume [49]. The patients of the current study received a restrictive fluid regimen and norepinephrine to avoid hypotension, as described previously [45].

Both hypoxia and hypercapnia strongly increase pulmonary vasoconstriction and contribute to PAH [50]. Severe ARDS per se is associated with profound hypoxia, which may be accompanied by hypercapnia [51]. Hypercapnia is the consequence of protective ventilatory strategies designed to reduce ventilator-induced lung injury. It also reflects increased dead space due to alveolar overdistension and ARDS severity [52].

Higher PEEP levels are frequently required in severe ARDS to avoid life-threatening hypoxia. However, transpulmonary pressure, despite low tidal ventilation when lung compliance decreases due to alveolar collapse, may be associated with increased end-inspiratory airway pressure [43]. Consequently, pulmonary capillaries become stretched and their caliber is reduced, resulting in increased pulmonary vasoconstriction [53,54].

By controlling arterial oxygenation and decarboxylation, even during ultraprotective ventilation [51], VV ECMO suppresses the major factors that increase pulmonary vascular resistance and cause PAH in severe ARDS, thereby sufficiently unloading the RV [55]. In this study cohort, VV ECMO was indicated to correct hypoxemia and hypercapnia, as well as allow ultraprotective ventilation, to prevent peak inspiratory (>27 cmH_2_O) and/or driving (>15 cmH_2_O) pressure (Supplemental Table S1). Although VV ECMO initiation in the current study resulted in adequate arterial oxygenation and normocapnia at a peak inspiratory and driving pressures < 25 cmH_2_O and < 10 cmH_2_O, respectively, PAH with or without RV failure persisted in these patients with severe ARDS.

Although inhaled NO in ARDS has been widely abandoned by intensivists because RCTs and meta-analyses have demonstrated no benefits for survival despite temporal improvements in oxygenation [29,30], it may be an option for decreasing RV afterload by lowering PAH [46]. Intriguingly, a positive response to inhaled NO was observed in only 30% of the patients of the current study. Similar findings were reported by Manktelow [56] on severe ARDS with septic shock. These studies were conducted before the widespread availability of VV ECMO and ultraprotective ventilator strategy. The data of the current study confirms the validity of this observation despite the use of current protective ventilation treatment regimens.

Inhaled NO use in ARDS has been widely studied over the last decades, and no evidence of direct NO toxicity has been observed at clinically relevant doses below 20 ppm [57]. However, conflicting evidence has been reported on whether inhaled NO contributes to increased acute kidney injury [58,59,60]. CKRT in this study did not indicate an increased rate of acute kidney injury during VV ECMO (Table 2). Thus, the potentially detrimental effects of inhaled NO on kidney function could not be confirmed in our study. Indeed, the rate of CKRT during VV ECMO and concomitant inhaled NO treatment reflects similar rates to those reported in other cohort studies on severe ARDS and VV ECMO without inhaled NO therapy [61].

The optimal dose and time of inhaled NO treatment in ARDS remains controversial. A European expert recommendation on the use of inhaled NO in adults with ARDS suggested that toxic side effects (e.g., met-hemoglobinemia and the formation of relevant nitrogen dioxide levels) are less likely when inhaled NO doses stay <20 ppm [57]. Initiating inhaled NO treatment as early as 24–72 h after the onset of ARDS has been suggested because inhaled NO is mainly effective during the early onset of ARDS. In this study, an average inhaled NO dose of 14.5 ppm was administered, and inhaled NO delivery was performed for a median duration of 3 days. Inhaled NO was initiated within 24 h after the diagnosis of either PAH and/or RV failure and inhaled NO was initiated during VV ECMO.

In terms of survival, persistent PAH and/or RV failure are known to contribute to worse outcomes such as in-hospital death, increased length in ICU, and a longer hospital stay. In this study, we found in-hospital mortality rates comparable to those reported in a recently published small single-center retrospective trial [62]. In a subgroup analysis, we found that the survival in inhaled NO treated patients differs between inhaled NO responders and non-responders, indicating that a NO responder might have a better survival rate. Of note, the inhaled NO cohort suffers from multiple comorbidities, all of which will worsen the clinical course of the patient regardless of inhaled NO administration (please refer to Table S2, which reports the Charlson Comorbidity Index for the study cohort). This assumption is further supported by the fact that the SAPS II and TISS scores in the inhaled NO group were higher at hospital discharge when compared to patients without NO treatment, indicating an increased severity of illness in this cohort.

A limitation of this study is the retrospective and monocentric nature of the analyses. The availability of RCTs involving patients with severe ARDS is limited after initial RCTs failed to demonstrate any beneficial effects of inhaled NO on survival and mortality. Also, retrospective analyses are difficult to control for an unbiased selection process of patients [63]. However, in this study all included patients were analyzed depending on treatment and thus were not subjected to selection bias for inhaled NO treatment. Lastly, a major limitation of this study is the small number of participants because patients with VV EMO with persistent PAH and/or RV failure treated with inhaled NO represent a very limited patient cohort.

## 5. Conclusions

In conclusion, this retrospective observational study suggests that persistent PAH and/or RV failure is associated with poor clinical outcomes in patients with severe ARDS and VV-ECMO support. Surprisingly, only 30% of the patient population responded to inhaled NO with a significant decrease in PAH and/or RV failure. We did not find an increased rate of continuous renal replacement therapy as a marker for acute renal failure when inhaled NO concentrations stayed below 20 ppm and when the duration of treatment was less than 3 days.

## Figures and Tables

**Table 1:** Baseline characteristics.

Characteristics	TOTAL (*n* = 366)	with Inhaled NO (*n* = 48)	without Inhaled NO (*n* = 318)	*p*-Value
**Demographics**				
age—(median (IQR)) [years]	56 (47–64)	57 (49–64)	55 (46–64)	0.6573
male sex—*n* (%) [male]	252 (69)	34 (71)	218 (69)	0.7505
weight—(median (IQR)) [kg]	90 (80–110)	95 (84–111)	90 (79–110)	0.0401
height—(median (IQR)) [cm]	175 (168–180)	177 (170–181)	175 (168–180)	0.2232
BMI—(median (IQR))	29 (26–35)	29 (27–38)	29 (26–35)	0.0416
**primary cause of ARDS**				
viral pneumonia—*n* (%)	124 (34)	20 (42)	104 (33)	0.6044
bacterial pneumonia—*n* (%)	63 (17)	6 (12)	57 (18)	
others—*n* (%)	179 (49)	22 (46)	157 (49)	
**indication for inhaled NO administration**				
pulmonary arterial hypertension (PAH)—no. [%]		23 (48)		
right heart failure (RVF)—no. [%]		17 (35)		
PAH and RV failure—no. [%]		8 (17)		

inhaled NO: inhaled nitric oxide. IQR: interquartile range. BMI: body mass index. ARDS: acute respiratory distress syndrome. PAH: pulmonary arterial hypertension. RV failure: right ventricular failure. *p* < 0.05 was considered to be significant.

**Table 2:** Clinical characteristics.

Characteristics	TOTAL (*n* = 366)	with Inhaled NO (*n* = 48)	without Inhaled NO (*n* = 318)	*p*-Value
**invasive mechanical ventilation**				
total time on mechanical ventilation—(median (IQR)) [days]	27 (14–48)	22 (13–40)	28 (14–49)	0.1550
Classified days on mechanical ventilation prior to ECMO—*n* (%)				
<48 h	185 (51)	25 (52)	160 (50)	
48 h–7 days	108 (30)	14 (29)	94 (30)	1.0000
>7 days	73 (19)	9 (19)	64 (20)	
tracheostomy—*n* (%)	153 (42)	18 (38)	135 (43)	0.5057
**ECMO**				
duration of ECMO support—(median (IQR)) [days]	12 (7–19)	14 (11–21)	11 (7–19)	**0.0363**
weaning failure from ECMO support—*n* (%)	177 (48)	35 (73)	142 (45)	**0.0003**
**adjunctive therapies**				
CKRT prior to ECMO—*n* (%)	107 (29)	16 (33)	91 (29)	0.5030
proning prior to ECMO—*n* (%)	136 (37)	17 (35)	119 (37)	0.9042
proning during ECMO—*n* (%)	193 (53)	28 (58)	165 (52)	0.7357
**Organ dysfunction**				
SOFA score at day 0 of ECMO initiation—(median (IQR))	9 (7–11)	9 (7–11)	9 (7–11)	0.7143
RESP score—(median (IQR))	0 (−3–2)	−1 (−3–2)	0 (−3–2)	0.9032
SAPS II score 24 h after ECMO initiation—(median (IQR))	47 (38–55)	44 (37–57)	47 (38–55)	0.7310
SAPS II score at discharge—(median (IQR))	49 (33–61)	57 (43–68)	46 (32–59)	**0.0037**
TISS score 24 h after ECMO initiation—(median (IQR))	28 (23–33)	29 (27–36)	27 (23–33)	0.1279
TISS at discharge—(median (IQR))	24 (14–31)	30 (20–37)	22 (12–30)	**0.0007**
no CPR prior to ECMO—*n* (%)	325 (89)	43 (90)	282 (89)	0.8531
sepsis—*n* (%)	70 (21)	5 (12)	65 (22)	0.1032

inhaled NO: inhaled nitric oxide. IQR: interquartile range. ECMO: extracorporeal membrane oxygenation. CRRT: continuous kidney replacement therapy. SOFA: sequential organ failure assessment. RESP: respiratory ECMO survival prediction. SAPS II: simplified acute physiology score. CPR: cardiopulmonary resuscitation. *p* < 0.05 was considered to be significant, and indicated by bold type.

## Data Availability

The clinical datasets generated and/or analyzed during the current study are not publicly available due to local data protection laws but are available from the corresponding author upon reasonable request.

## Supplementary Materials

The following supporting information can be downloaded at https://www.mdpi.com/article/10.3390/jcm13061555/s1, Table S1: Ventilation parameters and arterial blood gas analyses; Table S2: In-hospital survival analyses; Table S3: Known medical history indicated by the Charlson Comorbidity Index.
