# Supplementary material for: Analysis of Patients with Severe ARDS on VV ECMO Treated with Inhaled NO: A Retrospective Observational Study
Source: J Clin Med. 2024 Mar 8;13(6):1555. doi: 10.3390/jcm13061555 (PMC10970905; doi:10.3390/jcm13061555)
Supplement: Supplementary file 1 [file jcm-13-01555-s001.zip › jcm-2905163-supplementary.pdf]

Supplemental Material

Table S1. Ventilation parameters and arterial blood gas analyses						
characteristic	with inhaled NO (n=48)			without inhaled NO (n=318)		
	Day 1	Day 3	Day 7	Day 1	Day 3	Day 7
ventilation parameters						
minute ventilation – (median (IQR)) [l/min]	2.0 (1.5-3.1)	2.2 (1.8-3.7)	3.8 (2.8-4.8)	2.2 (1.5-3.2)	2.4 (1.6-3.5)	3.6 (2.3-5.9)
PEEP – (median (IQR)) [mbar]	20 (18-20)	20 (18-20)	18 (16-20)	18 (15-20)	18 (14-20)	18 (16-18)
peak inspiratory pressure – (median (IQR)) [mbar]	26 (25-28)	26 (25-28)	27 (26-28)	26 (25-28)	26 (24-28)	26 (24-28)
arterial blood gas analyses						
PaO_2 – (median (IQR)) [mmHg]	102 (100-105)	101 (79-124)	100 (85-113)	104 (102-105)	104 (102-116)	105 (101-113)
$PaCO_2$ – (median (IQR)) [mmHg]	38 (35-44)	39 (36-43)	42 (35-46)	37 (35-40)	40 (37-44)	38 (37-47)
pH – (median (IQR))	7.30 (7.3-7.4)	7.3 (7.3-7.4)	7.3 (7.3-7.4)	7.3 (7.3-7.4)	7.4 (7.3-7.4)	7.3 (7.3-7.4)
PaO_2/F_iO_2 – (median (IQR))	88 (67-109)	93 (75-111)	96 (78-131)	86 (66-107)	96 (79-121)	109 (88-155)
minimal F_iO_2 of a 24 hrs period (%)	80	70	70	70	65	50

inhaled NO: inhaled nitric oxide. IQR: interquartile range. PEEP: positive end-expiratory pressure. PaO_2: arterial partial pressure of oxygen. $PaCO_2$: arterial partial pressure of carbon dioxide. F_iO_2: fraction of inspired oxygen.

Table S2. In-hospital survival analyses				
characteristic	TOTAL (n=366)	with inhaled NO (n=48)	without inhaled NO (n=318)	p-value
outcome				
length of ICU stay – (median (IQR)) [days]	25 (13-47)	21 (13–35)	25 (13–49)	0.1900
length of hospital stay – (median (IQR)) [days]	27 (14– 53)	24 (14– 35)	27 (15–56)	0.1613
in-hospital death – n(%)	220 (60)	34 (71)	186 (58)	0.1036
median survival time – (median (IQR)) [days]	32 (13-379)	20 (12-43)	34 (18-385)	0.0590

inhaled NO: inhaled nitric oxide. ICU: intensive care unit. IQR: interquartile range. $p<0.05$ considered to be significant.

Table S3. Known medical history indicated by Charlson Comorbidity Index				
characteristic	TOTAL (n=366)	with inhaled NO (n=48)	without inhaled NO (n=318)	p-value
comorbidity				
myocardial infarction – n (%)	27 (7)	2 (4)	25 (8)	0.5543
congestive heart failure – n (%)	29 (8)	4 (8)	25 (8)	0.7814
peripheral vascular disease – n (%)	28 (8)	2 (4)	26 (8)	0.5577
cerebrovascular disease or TIA – n (%)	39 (11)	4 (8)	35 (11)	0.5759
dementia – n (%)	3 (1)	0 (0)	1 (3)	1.0000
COPD – n (%)	104 (28)	14 (29)	90 (28)	0.9015
Connective tissue disease – n (%)	8 (2)	0 (0)	8 (3)	0.6037
peptic ulcer disease – n (%)	10 (3)	2 (4)	8 (3)	0.6263
mild liver disease – n (%)	20 (5)	5 (10)	15 (5)	0.1609
moderate to severe liver disease – n (%)	6 (2)	0 (0)	6 (2)	1.0000
diabetes without end organ damage – n (%)	57 (16)	7 (15)	50 (16)	0.8391
diabetes with end organ damage – n (%)	28 (8)	3 (6)	25 (8)	1.0000
hemiplegia – n (%)	3 (1)	1 (2)	2 (1)	0.3449
moderate to severe CKD – n (%)	31 (8)	1 (2)	30 (9)	0.0999
solid tumor – n (%)	31 (8)	1 (2)	30 (9)	0.0999
leukemia – n (%)	7 (2)	3 (6)	4 (1)	0.0507
lymphoma – n (%)	10 (3)	0 (0)	10 (3)	0.3713
metastatic solid tumor	7 (2)	0 (0)	7 (2)	0.6010
AIDS – n (%)	2 (1)	0 (0)	2 (1)	1.0000
CCI	1 (0-2)	1 (0-2)	1 (0-2)	0.0511

inhaled NO: inhaled nitric oxide. CCI: Charlson Comorbidity Index. TIA: transient ischemic attack. COPD: chronic obstructive pulmonary disease. CKD: chronic kidney disease. AIDS: Acquired Immuno Deficiency Syndrome. $p<0.05$ considered to be significant.
